# Bacteria-induced morphogenesis of *Ulva intestinalis* and *Ulva mutabilis* (Chlorophyta): a contribution to the lottery theory

**DOI:** 10.1093/femsec/fix094

**Published:** 2017-07-14

**Authors:** Fatemeh Ghaderiardakani, Juliet C. Coates, Thomas Wichard

**Affiliations:** 1 School of Biosciences, University of Birmingham, Birmingham B15 2TT, UK; 2 Institute for Inorganic and Analytical Chemistry, Jena School for Microbial Communication, Friedrich Schiller University Jena, Lessingstraβe 8, 07743 Jena, Germany

**Keywords:** algal–bacterial interactions, axenic culture, *Bacteroidetes*, green macroalgae, *Microbacterium*, morphogenesis

## Abstract

The green marine macroalgae of the class Ulvophyceae (Ulvophytes) are common algae distributed worldwide particularly in intertidal areas, which play a key role in aquatic ecosystems. They are potentially valuable resources for food, animal feed and fuel but can also cause massive nuisance blooms. Members of Ulvaceae, like many other seaweeds, harbour a rich diversity of epiphytic bacteria with functions related to host growth and morphological development. In the absence of appropriate bacterially derived signals, germ cells of the genus *Ulva* develop into ‘atypical’ colonies consisting of undifferentiated cells with abnormal cell walls. This paper examines the specificity of bacteria-induced morphogenesis in *Ulva*, by cross-testing bacteria isolated from several *Ulva* species on two *Ulva* species, the emerging model system *Ulva mutabilis* and the prominent biofouler species *Ulva intestinalis*. We show that pairs of bacterial strains isolated from species other than *U. mutabilis* and *U. intestinalis* can fully rescue axenic plantlets generated either from *U. mutabilis* or *U. intestinalis* gametes. This laboratory-based study demonstrates that different compositions of microbial communities with similar functional characteristics can enable complete algal morphogenesis and thus supports the ‘competitive lottery’ theory for how symbiotic bacteria drive algal development.

## Teaser

Bacteria-induced morphogenesis of macroalgae.

## INTRODUCTION

Macroscopic marine algae (seaweeds) are significant primary producers in the oceans, which cover about 71% of earth's surface. Seaweeds are known as ‘ecosystem engineers’ due to their critical roles in marine environments, where they modulate the supply of resources to other species and alter the physical state of the surrounding environment, including sediments and water flow (Jones, Lawton and Shachak 1994; Alongi 1998). Seaweeds are important for maintaining local biodiversity (Schiel and Lilley 2007), create a protective environment for numerous invertebrate species (Wilson, Able and Heck 1990; Bulleri *et al.*^2002^) and provide an essential habitat for a range of epibionts, from microscopic organisms to macroinvertebrates (Fraschetti *et al.*^2006^). However, seaweeds can also cause significant nuisance blooms due to eutrophication in shallow coastal areas, which are detrimental to the environment and can harm ecosystems (Smetacek and Zingone 2013). In a commercial context, there is increasing interest in the use of marine biomass worldwide with multiple traditional and novel applications in food, fuel, high-value chemical and pharmaceutical industries and also in aquaculture, which is one of the promising market sectors (Kraan 2013).

There is growing interest in defining macroalgae-associated bacterial communities and macroalgal development and morphogenesis (Charrier, Coates and Stavridou 2017). A number of studies have shown that different species of seaweeds growing in the same ecosystem are associated with species-specific bacterial strains (Lachnit *et al.*^2009^, ^2011^; Barott *et al.*^2011^), leading to the hypothesis that the association between microorganisms and algae is host specific. This assumption is supported by observations that a significantly different phylum composition of bacteria was associated with each of three co-existing algae sampled at regular intervals over 2 years (Lachnit *et al.*^2011^). Moreover, the same species of seaweeds growing in different ecological habitats can associate with similar bacterial species (Lachnit *et al.*^2009^). Although it has been suggested that the bacterial–algal association is determined by the algal host (Longford *et al.*^2007^), bacterial isolates from seaweeds can vary with season and host life-cycle stage (Lachnit *et al.*^2011^) and even different tidal pools in close proximity (Burke *et al.*2011b). It was also reported by Cray *et al.* (2013) that the pre-eminence of some species, e.g. *Proteobacteria* and *Firmicutes*, is the result of their ability to compete with other species due to (i) high resistance to various stress factors (ii) and existence of different pathways for generating energy.

In contrast, based on a large-scale sequencing analysis, Burke *et al.* (2011a) suggested ‘the competitive lottery model’ for algal microbiomes, originally developed by Sale (1976), for explaining the coexistence of reef fish species in the same niche. They propose that different microbial communities with similar functional characteristics (defined by the genes present in the microbial genomes) can occupy the same algal species. Different microbiomes were isolated from different *Ulva australis* Areschoug samples in the same niche space and at different times in the year. The ‘competitive lottery’ model states that the structuring of microbial communities on the surface of host algae is controlled by the presence of particular microbial functional genes rather than microbial taxonomic entities (Burke *et al.*2011a). It is suggested that these functions are related to the ecophysiological roles of alga-associated microbial communities in general, i.e. detecting and moving towards the host, followed by attaching to the host and forming a biofilm, then responding to host environmental factors (Burke *et al.*2011b; Friedrich 2012). This functional assistance would result in formation of a holobiont, an entity composed of an alga with its associated functionally important bacteria (Egan *et al.*^2013^).

Growth and morphogenesis of various species of the green macroalaga *Ulva* such as *U. mutabilis*, *U. pertusa, U. linza* and *U. fasciata* can be controlled by a variety of marine bacterial species including members of the *Proteobacteria*, *Bacteroidetes* and *Firmicutes* (Provasoli 1958; Fries 1975; Nakanishi *et al.*^1996^; Matsuo *et al.*^2003^; Marshall *et al.*^2006^; Singh *et al.*^2011^; Spoerner *et al.*^2012^; Wichard 2015). Marshall *et al.* (2006) assessed the effects of 38 unique bacterial strains, isolated from three species of *Ulva*, on the growth rate and morphological development of *U. linza* axenic plantlets (treated with antibiotics) for 28 days. However, no single bacterium was able to completely restore normal morphology to axenic *U. linza*, in contrast to a recent observation in *U. mutabilis* applying bacteria isolated from *U*. *rigida* (Grueneberg *et al.*^2016^). Grueneberg *et al.* (2016) also demonstrated that *Ulva* can benefit from bacterial sources other than its own epiphytes, as diffusible waterborne morphogens can also affect *Ulva* development. This raises the question of specificity of the morphogen-producing bacteria.

To study microbial–algal interactions in the laboratory, strictly sterile (axenic) cultures of macroalgae pave the way for comparative research. Unlike other seaweeds, *Ulva* can be stably cultivated under laboratory conditions starting with axenic germ cells purified via their phototactic movement towards light, without applying antibiotics (Spoerner *et al.*^2012^; Vesty *et al.*^2015^; Wichard 2015; Weiss, Costa and Wichard 2017). The emerging model species *U. mutabilis* is routinely cultured in the laboratory with two bacteria, *Roseovarius* sp. strain MS2 (GenBank EU359909) and *Maribacter* sp. strain MS6 (GenBank EU359911), which confer proper morphogenesis. The *U. mutabilis* used in laboratory experiments is a fast growing and naturally occurring developmental mutant ‘slender’ of *U. mutabilis* was used (Alsufyani, Weiss and Wichard 2017). It shows only traces of the sea lettuce-like wild-type morphology and develops only primary rhizoids (Løvlie 1968; Wichard 2015). Axenic *U. mutabilis* cultures have an atypical ‘pincushion’ morphotype, in which a lack of holdfast and exterior cell wall distortions are the main characteristics. Bacterially derived substances govern rhizoid, cell wall and blade development (Spoerner *et al.*^2012^). Co-cultivation experiments using axenic gametes and MS2 revealed that this bacterium promotes cell division and algal blade cell growth, analogous to cytokinin function in land plants. A similar experiment using MS6 showed that MS6 induces formation of a proper cell wall and a primary rhizoid, analogously to auxin in land plants (Spoerner *et al.*^2012^; Wichard 2015). Overall, these morphogenesis-inducing bacteria secreted a variety (i.e. MS6- and MS2-like factors) of still uncharacterised morphogenesis-inducing factors (=morphogens) into the culture medium of *U. mutabilis* (Spoerner *et al.*^2012^; Weiss, Costa and Wichard 2017). The *U. mutabilis-Roseovarius-Maribacter* tripartite community established in the laboratory is an ideal model system with which to have controlled, repeatable conditions for further investigation of the interaction between a macroalga and its associated microbiome (Wichard *et al.*^2015^; Grueneberg *et al.* 2016).

Very few studies have systematically addressed the still unanswered question of the species specificity of epiphytic bacteria involved in the *Ulva*–bacterial interaction (Vesty *et al.*^2015^; Grueneberg *et al.*^2016^; Weiss, Costa and Wichard 2017) and defined the microbiome, starting from purely axenic cultures, which could affect various morphogenetic traits (Spoerner *et al.*^2012^; Vesty *et al.*^2015^). This study reports on a cross-testing of potentially morphogenesis-inducing bacteria, isolated from various *Ulva* species, between the model system *U. mutabilis* and *U. intestinalis*. Phylogenetic analysis suggested a very close relationship between *U. intestinalis* and *U. compressa* (Blomster, Maggs and Stanhope 1998; Hayden *et al.*^2003^) and also, in spite of the variation in morphologies and life cycles, between *U. mutabilis* and *U. compressa* (Løvlie 1964; Tan *et al.*^1999^; Wichard and Oertel 2010). Phylogenetically well-characterised bacterial strains, originally isolated by Marshall *et al.* (2006), were tested in a complementary bioassay, where test strains replaced first one, and then the other, bacterium in the tripartite *U. mutabilis*-*Roseovarius*-*Maribacter* community (Spoerner *et al.*^2012^; Wichard 2015).

## MATERIALS AND METHODS

### Algal samples

Vegetative and fertile *Ulva intestinalis* blades were collected three times between March 2015 and April 2016 from Llantwit Major beach, South Wales, UK (51°40΄ N; 3°48΄ W). The sampling site was composed predominantly of *Ulva* species of a uniform morphology, mixed with brown algae in places. Excess water and epiphytic species were removed at the site by blotting the sample's surface before storage on ice for transport back to the laboratory. This species cannot be reliably identified solely using morphological characteristics, and thus plastid-encoded *rbcL* (large unit ribulose bisphosphate carboxylase) and *tuf*A (plastid elongation factor) markers were used for identification (see below). Haploid gametophytes from the fast-growing tubular mutant of *U. mutabilis* named *slender* (sl-G(mt+)) (Føyn 1959; Løvlie 1964) were used for all cross-testing and comparative investigations with *U. intestinalis*.

### Genomic DNA extraction from *Ulva*, amplification and sequence analysis of *rbc*L and *tufA* genes

Genomic DNA was extracted from 25 mg seaweed samples using an ISOLATE II Genomic DNA Kit (Bioline, London, UK) according to the manufacturer's recommendations. DNA fragments of the *rbc*L and *tuf*A genes were amplified by PCR using 30 ng DNA and 1 μl VELOCITY DNA Polymerase (2 units /μl) (Bioline Ltd, UK) in a final volume of 50 μl per reaction according to the manufacturer's protocol. Two primer pairs were used for *rbc*L marker: (i) Forward—*rbc*LStart 5΄-ATGGCTCCAAAAACTGAAAC-3΄, Reverse - 750 5΄-GCTGTTGCATTTAAGTAATG-3΄ and (ii) Forward—F650 5΄- GAAAACGTAAACTCACAACC-3΄, Reverse— *rbc*LEnd 5΄-TTCTTTCCAAACTTCACA-3΄. The primers tested for *tuf*A marker were *tuf*A F 5΄-GGNGCNGCNCAAATGGAYGG-3΄, *tuf*A R 5΄-CCTTCNCGAATMGCRAAWCGC-3΄ (Famà *et al.*^2002^).

The PCR conditions were as follows: *rbc*l—an initial denaturation step at 94°C for 2 min, 29 cycles of 94°C for 45 s, 55°C annealing for 45 s and 90°C extension for 45 s, followed by a final elongation step at 72°C for 7 min; *tuf*A—an initial 4 min denaturation at 94°C, 38 cycles of 94°C for 1 min, 45°C annealing for 30 s, 72°C extension for 1 min, followed by 72°C final extension for 7 min (Saunders and Kucera 2010). PCR products were cleaned using the Thermo Fisher Scientific GeneJET PCR Purification Kit and sequenced on a capillary sequencer (ABI 3730, Applied Biosystems, USA) at the Functional Genomics Laboratory of the University of Birmingham.

The two primer pairs amplified two PCR products from the *rbc*L gene, 1–750 and 650–1430 (the 3΄ end) that overlapped, meaning a sequence for almost the entire gene could be obtained by sequencing and aligning the PCR products. PCR products were fully sequenced from both ends using the primers used to amplify them. The resulting sequences were aligned manually (there were no mismatches in the double reads for each PCR product) using the overlapping central 100 bp (650–750) to generate a consensus *rbc*L sequence (GenBank accession number MF038885). A single PCR product was generated for *tuf*A, which was sequenced from both ends. Alignment of the forward and reverse *tuf*A sequences demonstrated that they were identical, and a final consensus sequence of 772 bp was submitted to Genbank (MF162336).

The consensus sequences enabled the *Ulva* sample to be identified to species level by comparing the acquired sequence data with already available sequence data in GenBank by using a Basic Local Alignment Search Tool (BLASTN; Johnson *et al.*^2008^). Our sequences each had 100% match to only *U. intestinalis* samples.

### Cultivation conditions

The mutant slender (sl-G(mt+)) strain of *U. mutabilis* was propagated from unmated gametes derived from lab-grown parthenogenetic gametophytes. *Ulva intestinalis* was propagated from gametes derived from beach-collected gametophytes. All gametophytes were cultured in sterile culture flasks with gas-permeable screw caps (Nunc Int., Denmark) containing 100 mL *Ulva* Culture Medium (UCM; Stratmann, Paputsoglu and Oertel 1996) under the standard growth conditions including a 17:7 h light/dark regime at 18°C with an illumination of about 60 μmol photons m^−2^ s^−1^ provided by 50% GroLux, 50% day-light fluorescent tubes (Stratmann, Paputsoglu and Oertel 1996).

### Axenic cultures

Briefly, for preparation of axenic cultures, gametophytes of *U. mutabilis* and *U. intestinalis* were artificially induced to form gametangia by removal of at least two sporulation inhibitors (Stratmann, Paputsoglu and Oertel 1996; Vesty *et al.*^2015^). Afterwards, on the third morning in daylight, gametes were released from the gametangia by an additional medium change and removing the swarming inhibitor (Wichard and Oertel 2010). Freshly released gametes were purified from their accompanying bacteria by taking advantage of the gametes’ fast movement towards light through a narrow horizontal capillary under strictly sterile conditions in a laminar flow hood. This method was repeated at least three times to obtain axenic gametes. As final step, bacterial contamination was checked by plating a drop of the ‘gamete solution’ on Marine Agar plates (Roth, Karlsruhe, Germany, supplemented with 1% agar) and by PCR amplifications of the 16S rDNA in the supernatant (Spoerner *et al.*^2012^; Wichard 2015).

### Bacterial strains

By using axenic gametes in a standardised bioassay, it is possible to determine which microbes induce the algal morphogenesis through morphogenetically active substances (morphogens) (Grueneberg *et al.*^2016^). A large collection of *Ulva*-associated bacteria was available, isolated by the Callow laboratory (Marshall 2004; Marshall *et al.*^2006^). These bacterial strains isolated from multiple *Ulva* species (including *U. linza*, *U. lactuca*, *U. compressa* and *Enteromorpha* sp.) have been maintained at –80°C in glycerol as source cultures since collection: not all have been previously assigned a genus (Marshall 2004; Marshall *et al.*^2006^; J. Callow unpublished; Table 1). UL19, EC19, UL16, EC34, E1 and UL2 were selected, which induced a wide range of degrees of growth of axenic *Ulva* plantlets (based on Marshall *et al.*^2006^ or our preliminary tests; Tables 1 and 2).

**Table 1.** Previous data for bacterial strains chosen for this study.

Isolate ID	Original Genbank accession number	Algal host*^a^*	Morphology score*^b^*	Closest matching strain in GenBank after collection	% Sequence similarity
UL19	AM180742	*Enteromorpha compressa*	2	*Shewanella gaetbuli*	96
EC19	Not previously identified	*Enteromorpha compressa*	–	Not previously identified	–
E34	Not previously identified	*Enteromorpha sp.*	–	Not previously identified	–
E1	Not previously identified	*Enteromorpha sp.*	–	Not previously identified	–
UL16	AM180741	*Ulva linza*	3	*Cellulophaga* sp*.*	92
UL2	AM180737	*Ulva linza*	1–2	*Bacteroidetes bacterium*	94

*^a^*Callow JA, unpublished data. *^b^*‘Morphology after 28 days assessed on a semi-quantitative scale. 0: Little tubular growth (G10) from central callus of *Ulva linza*; 1: 10–30 tubular extensions; 2:30–50 tubules; 3: 950 well-developed tubules’ (Marshall *et al.*^2006^).

**Table 2.** Current classification of bacteria isolated from three *Ulva* spp plus summary of new data in this paper.

Isolate ID	Phenocopy of?	Closest matching strain in GenBank	Phylum	New Genbank Accession No#	% Sequence similarity
UL19	Axenic	*Microbacterium* sp.	*Actinobacteria*	KY827088	100
EC19	MS6	*Microbacterium* sp.	*Actinobacteria*	KY827089	99
E34	MS2	*Paracoccus* sp.	*Alpha-proteobacteria*	KY827090	99
E1	Axenic	*Planococcus* sp*.*	*Firmicutes*	KY827091	99
UL16	MS2	*Cellulophaga* sp*.*	*Bacteroidetes*	KY827092	99
UL2	MS2	*Paracoccus* sp.	*Alpha-proteobacteria*	KY827093	99

### Phylogenetic characterisation of bacteria

Ten microlitres of each of bacterial isolate was cultivated in 10 mL Marine Broth (MB; Roth, Karlsruhe, Germany) and then directly streaked onto Marine Agar plates to obtain single colonies. The plates were incubated at 20°C for 5 days, then distinct colonies were picked off and transferred with a sterile loop into new bottles containing 10 mL MB. Bacterial DNA was extracted according to the manufacturer's instructions using a DNeasy Blood and Tissue kit (Qiagen, Hilden, Germany). To identify, or re-classify, the identity of the six bacterial strains using to up-to-date classifications, partial 16S rDNA sequences (∼1500 bp) were amplified from these strains using the primer pair 27f (GGG TTT GAT CCT GGC TCA G) and 1390r (ACG GGC GGT GTG TRC AA) (Olsen *et al.*^1986^; Burggraf *et al.*^1992^). The reaction master mix contained 2.5 μL of PCR buffer 10% (100 mmol L^−1^ Tris/HCl pH 8.3, 500 mmol L^−1^ KCl, 15 mmol L^−1^ MgCl_2_), 1.25 μL of BSA (20 mg/mL), 1 μL each of forward and reverse primer (20 mM), 0.5 μL dNTPs 100 mM (dATP, dCTP, dGTP, dTTP), 0.15 μL Taq polymerase (5 units/μl) and ∼100 ng of template DNA. The PCR protocol included a 5-min initial denaturation at 95°C, followed by 31 cycles at 95°C for 30 s, 58°C for 30 s, 72°C for 90 s, finally 1 cycle of 7 min at 72°C and storage at 4°C. PCR products were then subjected to forward primer sequencing using the chain termination method (GATC, Göttingen, Germany). The closest homologous sequences in the GenBank database were recorded in Table 2. Two isolates belonged to the phylum *Proteobacteria* (*Alphaproteobacteria* class), two to the phylum *Actinobacteria*, one to the phylum *Bacteroidetes* and one belonged to the phylum *Firmicutes* (Table 2).

### Bioassay-guided testing of algal morphogenesis inducing bacteria associated with *Ulva*

To survey the activity of potentially morphogenesis-inducing bacteria, the ‘*Ulva* bioassay array’ based on a multiwell plate (96 wells) format was used (Spoerner *et al.*^2012^; Grueneberg *et al.*^2016^).

To avoid any bias and thus minimise variation between several experimental set ups that would make the results ambiguous, positive and negative controls were run on identically prepared 96-well plates at the same time. As positive controls, *U. mutabilis* axenic gametes were incubated with the well-characterised *Roseovarius* sp. strain MS2 alone, *Maribacter* sp. strain MS6 alone and MS2+MS6 (triplicates of each) (as in Spoerner *et al.*^2012^; the taxonomy of MS2 and MS6 were recently reclassified by Grueneberg *et al.*^2016^).

The same treatments were also carried out with axenic gametes of *U. intestinalis*. As a negative (axenic) control, 12 wells in one row were left without any bacterial inoculation in each plate. For further comparison and evaluation, *U. intestinalis* was grown in flasks with the normal complement of *U. intestinalis*-associated bacteria by using non-purified gametes. Three biological replicates were conducted in parallel for each experiment.

The stock solution of freshly prepared axenic gametes was diluted with UCM to obtain the optimum concentration of gametes (about 300 gametes/mL). The density of gametes in the axenic stock solution was measured by flow cytometry (BD Accuri® C6) by comparing gamete samples to standards provided by the manufacturer (BD Biosciences, New Jersey, USA). The gamete solution was distributed in 96-well multiwell plates, 100 μL in each well. After incubation of plates overnight at room temperature in darkness, gametes homogenously settled down to the bottom of plates.

**Figure 1.**
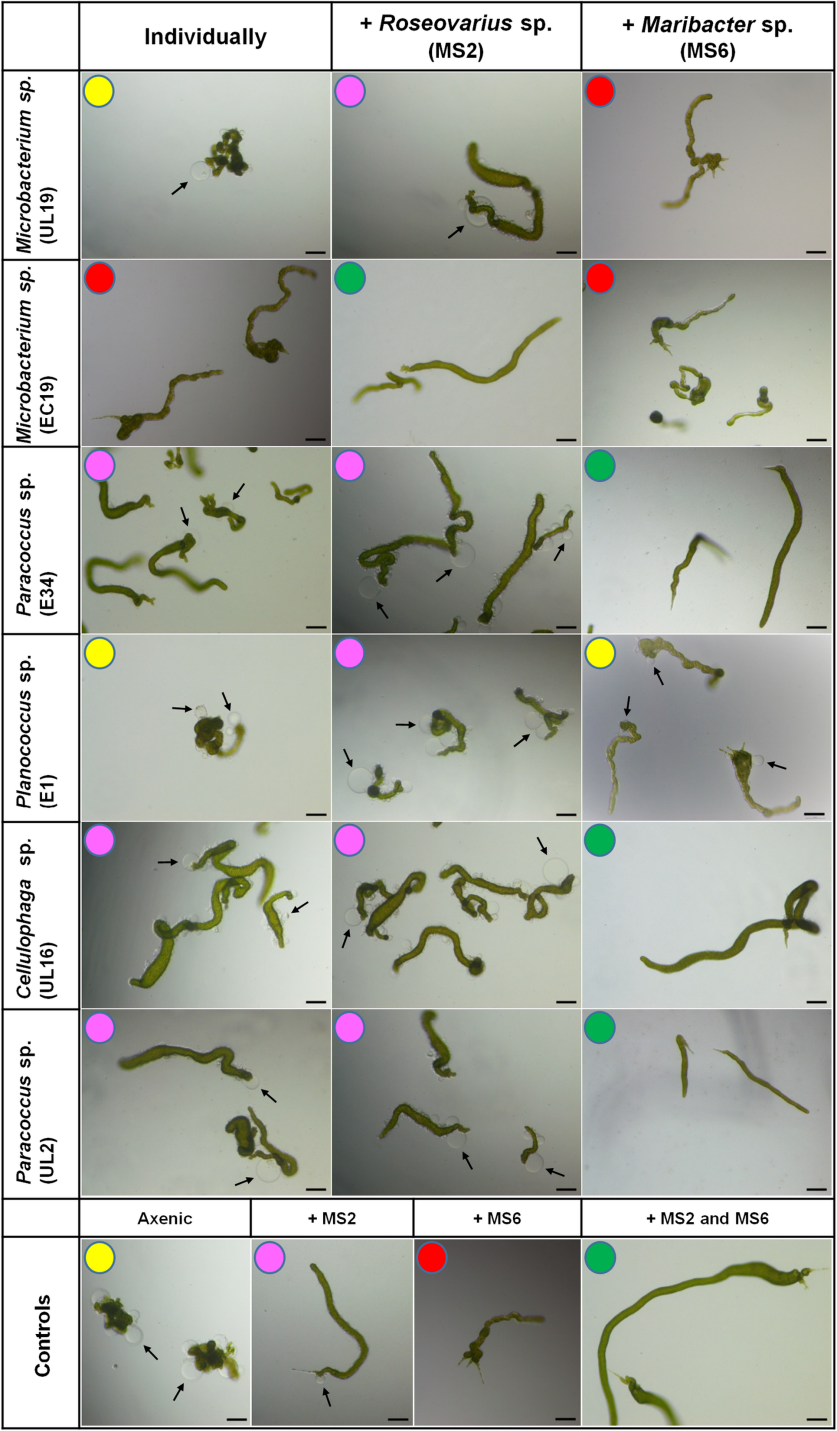
Morphogenesis assessment of *U. mutabilis* using the ‘*Ulva* bioassay array’. The multiwell-based testing system of morphogenetic activity using axenic gametes of *U. mutabilis* allows the fast determination of the different morphotypes induced by bacteria isolated from various *Ulva* species, singly and in pairwise combination with the bacteria *Roseovarius* sp. MS2 and *Maribacter* sp. MS6. Representative morphotypes are categorised by a color code: Yellow circle (axenic morphotype): callus-like cultures with typical colorless cell wall protrusions. Magenta circle (morphotype induced by the MS2-like factor)**:** germlings with normal cell division towards one direction but still covered by protrusions and differentiated rhizoid cells are missing. Red circle (morphotype induced by the MS6-like factor)**:** plantlets show a proper cell wall and rhizoid formation but the blade does not develop. Green circle (completely recovered morphotype)**:** characteristic usual morphotype with normal blade and rhizoid formation. Propagules are 3 weeks old. Controls are shown in the bottom row. Arrows indicate the typical colourless protrusions from the exterior cell walls of axenic cultures. Scale bars = 100 μm.

**Figure 2.**
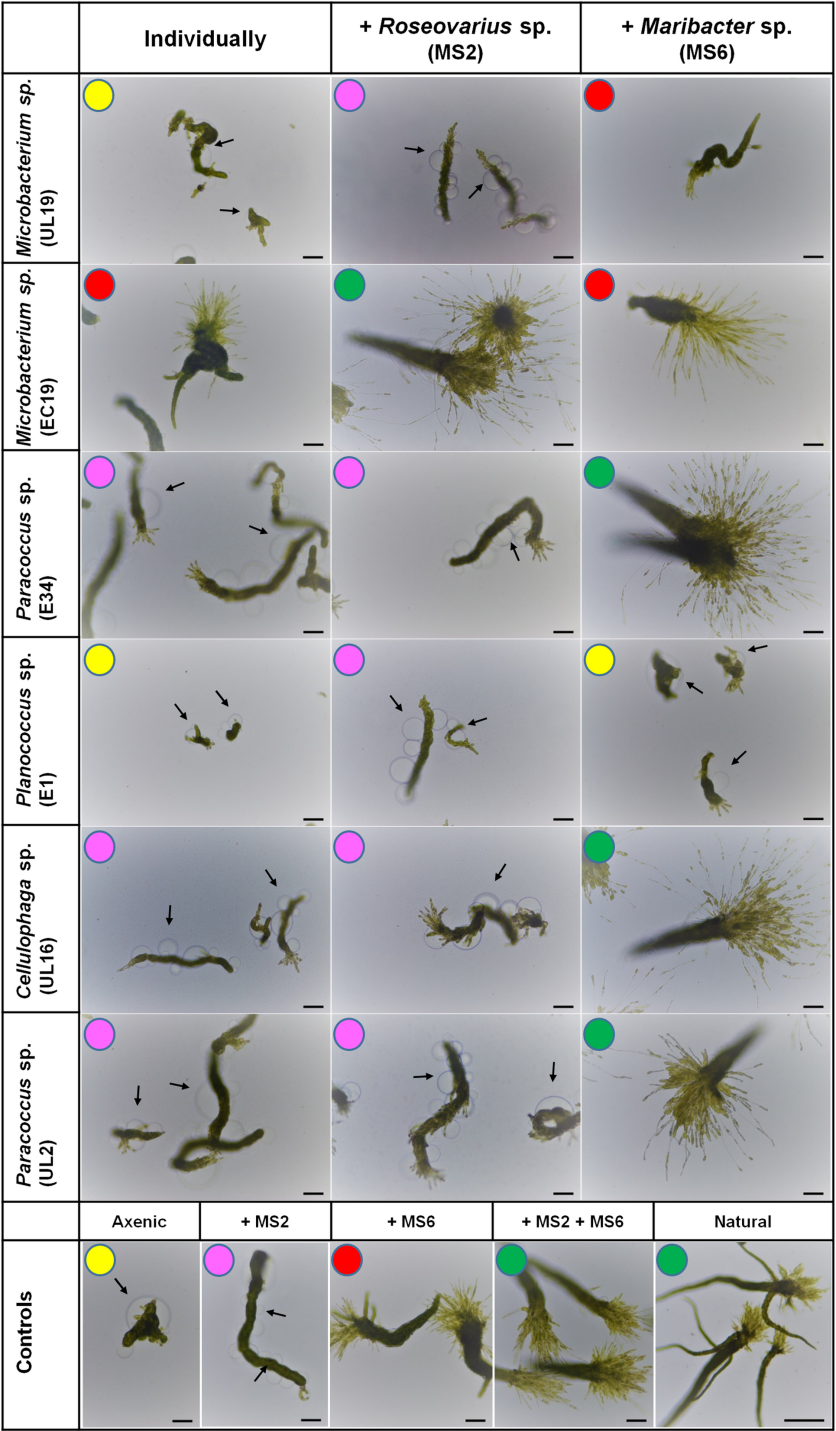
Morphogenesis assessment of *U. intestinalis* using the ‘*Ulva* bioassay array’. Different morphotypes of *U. intestinalis* induced by bacteria isolated from various *Ulva* species singly and in pairwise combination with the bacteria *Roseovarius* sp. MS2 and *Maribacter* sp. MS6*.* Arrows indicate the typical colourless protrusions from the exterior cell walls of axenic cultures. Representative morphotypes are categorised by the same colour code as described in Fig. 1. Propagules are 3 weeks old. Controls are shown in the bottom row. There was no significant differences in growth and morphology between propagules grown in the presence of the strains MS2 and MS6 compared to those grown in the presence of the natural microbiome (‘Natural’). Scale bars = 100 μm.

**Figure 3.**
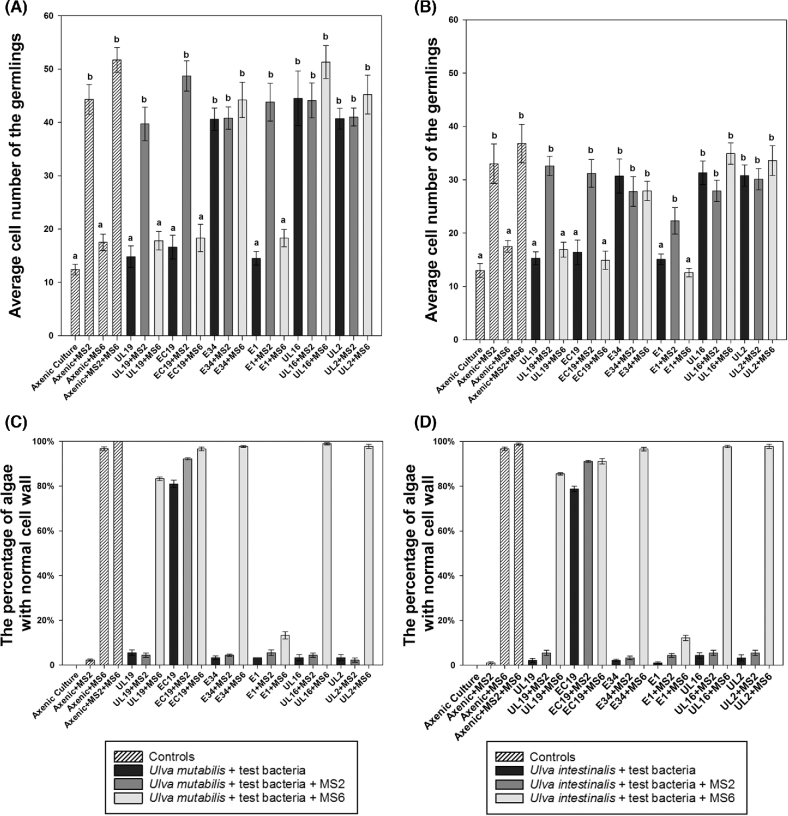
Semi-quantitative data of bacteria-induced growth and morphogenesis derived from the ‘*Ulva* bioassay array’ with axenic *U. mutabilis* (**A**, **C**) and *U. intestinalis* (**B**, **D**) gametophytes. (A and B) To estimate the activity of the MS2-like factor, the total cell numbers in thalli of *U. mutabilis* (A) and *U. intestinalis* (B) plantlets were counted 10 days after inoculation with *Microbacterium* sp. EC19, *Microbacterium* sp. UL19, *Planococcus* sp. E1, *Paracoccus* sp. E34, *Cellulophaga* sp. UL16 or *Paracoccus* sp. UL2. Controls show the morphogenetic activity on gametes without bacteria, with the bacterial strain MS2, with the bacterial strain MS6 and with both MS2 and MS6 bacterial strains. (C and D) To determine the activity of the MS6-like factor, the proportion of thalli of *U. mutabilis* (C) and *U. intestinalis* (D) with normal cell wall development was evaluated as a percentage of total thalli 10 days after inoculation with bacteria listed above. A one-way ANOVA was performed to reveal statistically significant differences, followed by a Dunn's multiple comparison test to determine which groups differ (*P* < 0.05), indicated by the letters a and b. Error bars represent (A, B) confidence intervals (*P* = 0.95; n > 30 individual algae) or (C, D) standard deviations (n > 30 individual algae).

To observe the morphogenetic effects of *Ulva*-associated bacteria, *U. intestinalis* and *U. mutabilis* (*slender*, gametophyte, mt[+]) axenic gametes were inoculated with the bacteria isolated from three different *Ulva* species and *U. mutabilis*, individually and in combinations (triplicates of each) (Figs 1–3) as recently established by Weiss, Costa and Wichard (2017). Bacterial strains were grown in marine broth for 3–7 days depending on the strain. The optical density (OD) of the bacteria was measured and each strain was diluted in UCM to an OD of 1.0 and then serially diluted in additional UCM to OD of 10^−4^. Ten microlitres of this ‘stock’ solution was then added to 100 μl of UCM containing *Ulva* gametes in a multiwell plate, giving a final calculated OD of 10^−5^. The same ‘pattern’ of bacterial strains was used on each plate, with plates growing under homogeneous light conditions and controlled temperature (Stratmann, Paputsoglu and Oertel 1996). Up to five technical repeats were carried out for each of three biological repeats—in each biological repeat, each plate was in a different position in the growth chamber, reducing the risk of ‘pseudo-replication’. To avoid any contamination, plates were covered with gas permeable sealing film (Breathe-Easy, Diversified Biotech, MA, USA) and transferred to growth chamber under standard conditions (Wichard and Oertel 2010). Over the next 3 weeks, plantlets were observed under the inverted microscope (DM IL LED, Leica, Wetzlar, Germany). The qualitative features considered under microscopic observation included the presence of unusual cell wall protrusions (‘bubble-like’ structures), thallus length and differentiated rhizoid cells (Spoerner *et al.*^2012^). Quantification of the average blade cell number and the percentage of thalli with entirely normal cell walls were carried out. Cell numbers were compared using one-way analysis of variance (ANOVA), with a Dunn's multiple comparison posteriori test using SigmaPlot 13 software (Systat Software, San Jose, CA). Comparison of the activities of MS2 and MS2-like bacteria between *U. mutabilis* and *U. intestinalis* were compared using two-way ANOVA followed by a Dunn's multiple comparison test using SigmaPlot software.

## RESULTS

### Bioassay-guided classification of the bacteria-induced morphogenesis of *Ulva mutabilis*

As demonstrated by Spoerner *et al.* (2012), axenic *U. mutabilis* plants develop a characteristic morphology with a lack of holdfast and distortions of the exterior cell wall (Fig. 1). The effect of six individual bacterial species isolated from *Ulva* species was assessed for their ability to ‘rescue’ the morphology of axenic *U. mutabilis* gametes back towards the complete non-axenic state (Fig. 1). A range of different morphotypes were stimulated by the individual bacterial strains, but none of them could solely elicit complete algal morphogenesis and normal development of *U. mutabilis* (Fig. 1).

Various *Ulva* bacterial isolates were able to promote marked morphological changes in *U. mutabilis*. Three out of these four isolates, *Paracoccus* sp., strains E34 and UL2, as well as *Cellulophaga lytica* UL16 caused cell divisions, like the reference strain *Roseovarius* sp. MS2 (Fig. 1). As previously observed, the release of the MS2-like factor was not genus dependent (Spoerner *et al.*^2012^; Grueneberg *et al.*^2016^). Although in previous studies the MS2-like factor was frequently assigned to genera from the *Alphaproteobacteria*, we now show that the specific morphogenetic activity of blade induction can also be carried out by *Cellulophaga* sp. (Fig. 1; Table 1).

However, as the MS2-like factor does not drive normal cell wall development and protrusions remained visible (Fig. 1), further bacteria are necessary to complement the functional traits and to complete *Ulva*'s morphogenesis. We show that the *Actinobacterium Microbacterium* sp. EC19 possesses this activity and can induce both cell differentiation and cell wall formation, but failed to induce a proper blade, which is analogous to the activity of the reference strain MS6 (Fig. 1). The two other tested bacteria *Microbacterium* sp. UL19 and *Planococcus* sp. E1 had no distinct effect on the growth and morphology of *U. mutabilis* and at the end of the experiment, algae cultured with these bacteria resembled axenic controls (Fig. 1). In addition, the strain E1 seems to negatively interfere with MS6, as the typical morphogenetic activities of MS6 are not visible in the presence of E1 (Figs 1 and 2). Overall, this shows that the morphogenetic activity of bacteria towards *U. mutabilis* is bacterial strain specific rather than correlating with bacterial genus.

### Bioassay-guided classification of the bacteria-induced morphogenesis of *Ulva intestinalis*

To address the question of how *Ulva* species-specific the morphogenetic activities of bacteria are, axenic cultures *of U. intestinalis* were prepared through application of the methods originally developed for *U. mutabilis*. In the absence of epiphytic bacteria, *U. intestinalis* plantlets reverted to an undifferentiated callus of cells (Fig. 2, controls), similar to axenic plantlets of *U. mutabilis* (Spoerner *et al.*^2012^; Vesty *et al.*^2015^) with unusual colourless protrusions from the exterior cell wall instead of the normal tubular morphology (Fig. 2, controls). As observed for *U. mutabilis*, the mode of action of *Paracoccus* sp. E34, *Cellulophaga* sp. UL16 and *Paracoccus* sp. UL2 on *U. intestinalis* plantlets was indistinguishable from the activities of the control reference strain MS2 (compare Fig. 1 and Fig. 2). The same was true for the respective activity of *Microbacterium* sp. EC19. Under the influence of EC19, axenic gametes of the ‘slender’ mutant develop into minute short rows of degenerated blade cells with normal cell walls and rhizoid formation. EC19 thus revealed similarity to the activity of the MS6-like factor with *U. intestinalis* in addition to its activity with *U. mutabilis* (Fig. 2, compare with the MS6 control). The strong effect on rhizoid formation was prominent, forming multiple secondary rhizoids (Fig. 2).

### Semi-quantification of the morphogenesis inducing activity of bacteria

For further evaluation, a more detailed analysis was conducted. The number of cells produced by developing *Ulva* plantlets (Fig. 3A and B) and the degree of formation of cell wall protrusions as a result of a lack of MS6 morphogens was determined (Fig. 3C and D). Upon the inoculation of axenic gametes of *U. mutabilis* with the strains E34, UL16 or UL2, the average cell numbers increased 4-fold (Fig. 3A; *P* < 0.05) within 2 weeks: these strains were therefore as active as the reference strain MS2. There was no significant difference between the activity of MS2 and the MS2-like bacteria E34, UL2 and UL16 on *U. mutabilis*: all bacteria can rescue the cell division to the same degree (Fig. 3). However, two-way ANOVA revealed that the morphogenetic activity of the bacteria E34, UL16 and UL2 was significantly lower on *U. intestinalis* (Fig. 3B; *P* < 0.05) than on *U. mutabilis* (Fig. 3A; *P* < 0.05) within the 2-week bioassay. Overall, we conclude that differences in growth of both algae are due to slower growth rates of *U. intestinalis* compared to *U. mutabilis* rather than the mode of action of the factors released by the respective bacteria.

### A new tripartite system established with *Ulva intestinalis* and *Ulva mutabilis*

**Figure 4.**
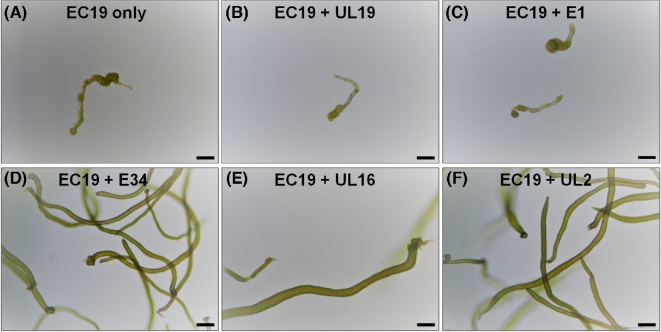
Establishment of a tripartite community of *U. mutabilis* with novel bacteria. Three-week-old *U. mutabilis* gametophytes are shown inoculated with bacteria isolated from different *Ulva* species in pairwise combination. Axenic gametes of *U. mutabilis* were inoculated with (**A**) *Microbacterium* sp. EC19 only, and together with (**B**) *Microbacterium* sp. UL19, (**C**) *Planococcus* sp. E1, (**D**) *Paracoccus* sp. E34, (**E**) *Cellulophaga* sp. UL16 or (**F**) *Paracoccus* sp. UL2. (**D-F**) Due to the complementary functional traits of the bacteria, the tripartite community can completely recover the morphogenesis of *U. mutabilis*, whereas the bacterial isolates UL19 and E1 do not contribute to the algal development. The bioassay system was scaled up using sterile culture flasks. Scale bars = 100 μm.

**Figure 5.**
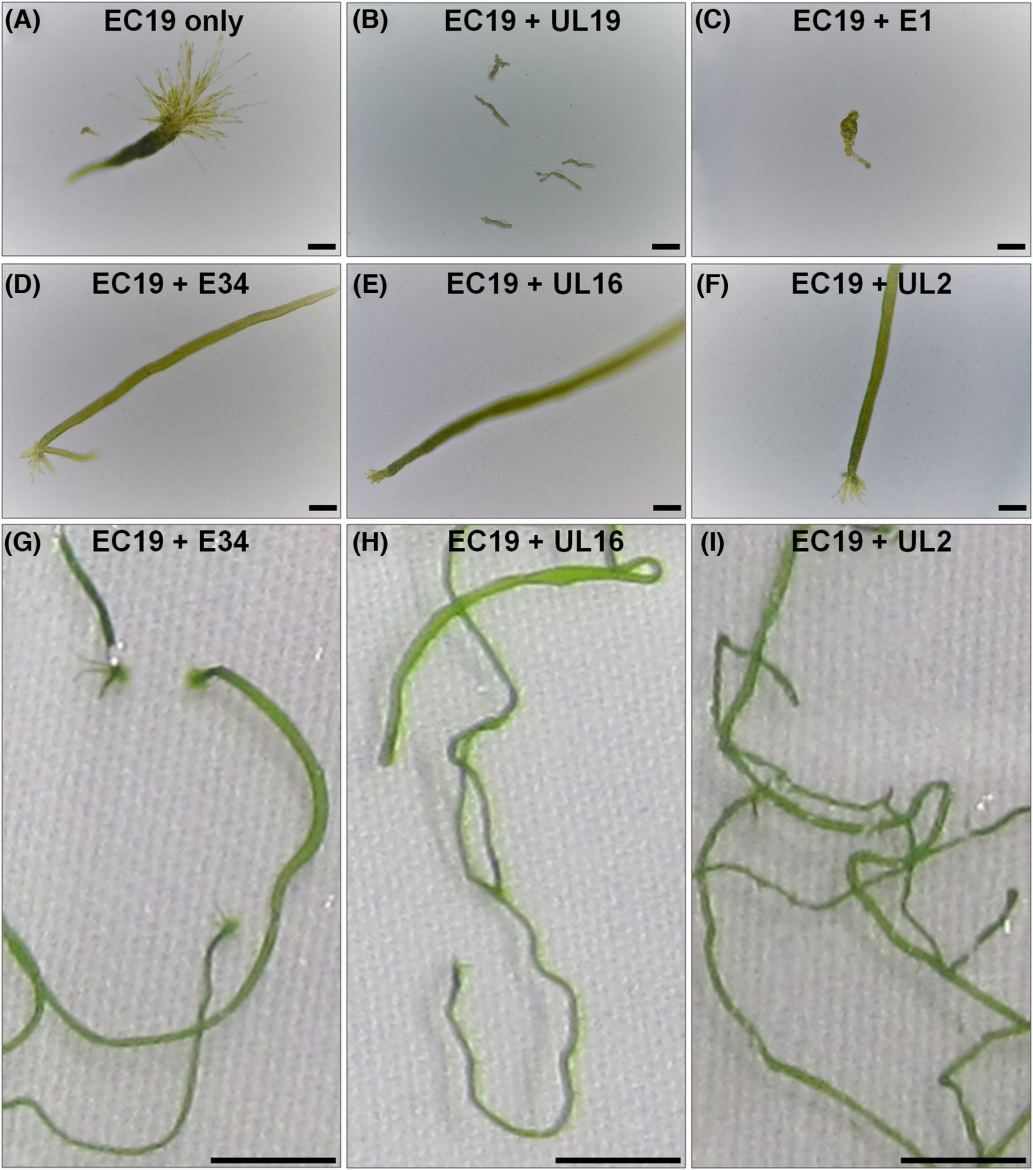
Establishment of a tripartite community of *U. intestinalis.* Three-week-old *U. intestinalis* gametophytes are shown inoculated with bacteria isolated from different *Ulva* species in pairwise combination. Axenic gametes of *U. mutabilis* were inoculated with (**A**) *Microbacterium* sp. EC19 only, and together with (**B**) *Microbacterium* sp. UL19, (**C**) *Planococcus* sp. E1, (**D**) *Paracoccus* sp. E34, (**E**) *Cellulophaga* sp. UL16 or (**F**) *Paracoccus* sp. UL2. (**D-F**) Due to the complementary functional traits of the bacteria, the tripartite community can completely recover the morphogenesis of *U. intestinalis*. (**G**–**I**) The thallus of *U. intestinalis* continues growing under these conditions and increases significantly in size within one more week. (A–F) Scale bars = 100 μm and (G–I) scale bars = 1 cm.

The applied strains have been tested in previous studies with *U. linza* and bacterial activities were classified according morphological scores by Marshall *et al.* (2006) (Table 1), but different functional traits for growth and morphogenesis were not determined at that time. Therefore, in our study, bacterial strains were selected according to their two main functional traits (Figs 1 and 2) in order to define new tripartite communities with *U. mutabilis* (Fig. 4) or *U. intestinalis* (Fig. 5). Importantly, there was no species-specificity between *U. intestinalis* and *U. mutabilis*, because a range of bacteria can perform their eco-physiological functions similarly in both species (Figs 1 and 2).

**Figure 6.**
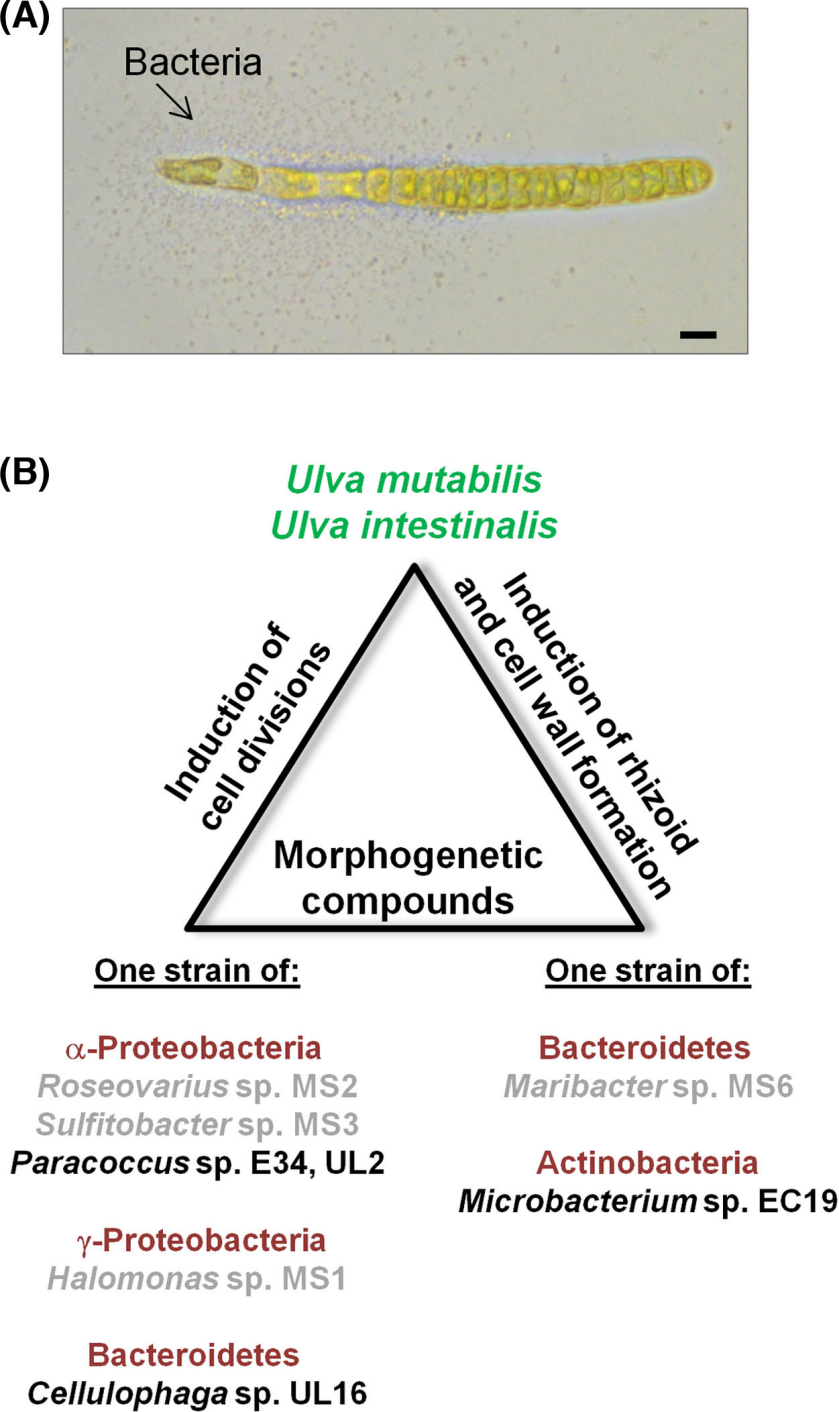
Model systems for bacteria–macroalgae interactions. (**A**) Bacterial biofilm formation upon inoculation of *U. intestinalis* axenic gametes with *Microbacterium* sp. EC19 and *Paracoccus* sp. E34 for 5 days. Bacteria concentrate around the rhizoid. Scale bars = 10 μm. (**B**) Effects of a defined microbiome can be reliably tested using tripartite systems of *U. mutabilis* or *U. intestinalis* and multiple combinations of algal morphogenesis-inducing bacteria. Figure was adapted and changed from Grueneberg *et al.* (2016). Names of bacterial strains, which were tested in this study for the first time, are printed in black.

The morphogenesis of *U. intestinalis* and *U. mutabilis* axenic germlings completely recovered in co-cultivation experiments with *Microbacterium* sp*.* EC19, the only selected strain that could phenocopy the *Maribacter* sp. MS6, and in combination with any one of E34, UL16 or UL2, which phenocopy the *Roseovarius* sp. MS2 (Figs 4 and 5). Upon inoculations, bacteria grew and formed a cluster around the rhizoid of *U. intestinalis* (Fig. 6A) resembling the tripartite *U. mutabilis-Roseovarius-Maribacter* system (Spoerner *et al.*^2012^). It is not clear whether a single or both bacterial species are present at the rhizoid or how they achieve this, as only some species of *Microbacterium* sp. EC19 and *Paracoccus* sp. E34 are motile (Kelly, Rainey and Wood 2006). Starting with this biofilm, *U. intestinalis* continues growing in the presence of any of the specifically designed microbiomes (Fig. 5G–I). In summary, a newly standardised *U. intestinalis* tripartite system has been established with various pairs of bacterial symbionts isolated from multiple *Ulva* species (Fig. 6).

## DISCUSSION

This study, starting with axenic cultures, has shown that phylogenetically distinct bacteria isolated from *Ulva* species other than *Ulva mutabilis* possess morphogenetic activity and can be used in combination to set up a tripartite system in an established model and phenocopy the reference strains MS2 and MS6. We have also shown that that the economically important *U. intestinalis* can function similarly in a tripartite system. We have defined new ‘minimal’ microbiomes that promote growth, development and morphogenesis in *U. mutabilis* and *U. intestinalis*. The morphogenetic activity of all positively tested bacterial strains was comparable with the activity found in sterile-filtered natural water samples collected from the lagoon Ria Formosa (Portugal) using the same standardised bioassay (Grueneberg *et al.*^2016^).

This is the first report demonstrating the activity of an MS2-like factor within the phylum *Bacteroidetes*. Although experiments with boiling extracts of the *Maribacter* sp. MS6 revealed that this strain produces an MS2-like factor as well, the morphogenetic compound is not released into the environment (Spoerner *et al.*^2012^). In any case, it should be taken into account that different compounds could show similar ecophysiological activities on *Ulva*'s morphogenesis. Our data contrast with Grueneberg *et al.* (2016), who also reported two isolates, *Algoriphagus* sp. and *Polaribacter* sp., that could each singly rescue complete morphology in *U. mutabilis*. Our study reveals again that strains of the same genus, UL19 and EC19, can harbour different functional traits.

Until now, only very few *Actinobacteria* have been tested on *Ulva* species for their effect on algal morphogenesis (Marshall *et al.*^2006^), and *Microbacterium* sp. EC19 is the first representative of this phylum with a defined activity to *U. mutabilis* and *U. intestinalis*. Interestingly, the phylum *Actinobacteria* was also one of the major beneficial bacterial phyla detected on *Gracilaria vermiculophylla* from the North Sea (Lachnit *et al.*^2011^) and associated with *Laminaria* populations (Wiese *et al.*^2009^; Salaün *et al.*^2010^).

### Host specificity of epiphytic bacteria on *Ulva* species, or lottery theory?

This study tested whether a consistent core community is necessary to drive complete morphogenesis of *Ulva* species or whether a range of bacterial isolates can phenocopy the algal phenotypes induced by the strains MS2 (*Roseovarius*) and MS6 (*Maribacter*).

Large-scale 16S rRNA gene sequencing of the bacterial populations present on various individual of *U. australis* demonstrated that a consistent core microbiota could not be detected, and a large number of bacterial individuals are able to colonise the algal surfaces (Burke *et al.*2011a,b). The temporal and spatial comparisons carried out by Tujula *et al.* (2010) have revealed that the microbiota on *U. australis* varies considerably among the individuals collected from both the same, and three different, tidal pools and also over different seasons. Despite these considerable shifts, it also has been demonstrated that a set of bacterial epiphytes belonging to *Alphaproteobacteria* and *Bacteroidetes* remained stable over space and time, implying their possible significant role in function of this bacterial community (Tujula *et al.*^2010^). However, bacteria belonging to the less-abundant phylum *Actinobacteria* on *Ulva*'s surface (Friedrich 2012) can harbour strong (morphogenetic) effects on algal growth as demonstrated in our study.

Bioassays testing bacteria-induced morphogenesis, starting with axenic cultures, provide a unique approach to assess the specificity of bacterial functional traits within bacteria–macroalga interactions. Some evidence suggested that the activities of the strain MS6, promoting rhizoid growth and normal cell wall development, were rare (Grueneberg *et al.*^2016^), in contrast to the activity of strain MS2, which promotes growth and blade development. Therefore, the MS6-like factor was considered to be a genus-specific functional trait, due to the fact that those marine bacteria are hard to culture (Wichard 2015; Grueneberg *et al.*^2016^). With the findings of the current study, we show for the first time that both functional traits can be delivered by more than one bacterial phylum. The tripartite community of *Ulva* and bacteria can be established as long as certain bacteria release compounds with cytokinin-like activity, whereas others provide an auxin-like activity (Fig. 6). Overall, our data support the competitive lottery hypothesis (Sale 1976; Burke *et al.*2011a), which implies that waterborne morphogenetic compounds are provided by various bacteria within a specific niche (algal surface). This seems to be a random process, which is based on the presence of functional genes and their functional characteristics rather than on a requirement for bacteria to belong to particular taxonomic groups. Our study shows that in the laboratory, two species of green algae can use combinations of compounds derived from multiple species of bacteria to drive their correct morphogenesis, and we hypothesise that similar situations may arise in their natural environment, where algae are exposed to multiple bacteria and waterborne compounds.

## CONCLUSIONS

Designed microbiomes were used to test the algal morphogenesis-inducing traits of bacteria in both the standard test strain *Ulva mutabilis* and a new algal species, *U. intestinalis*. By adding different bacteria singly or in pairs to *Ulva* gametes, our bioassays revealed that (i) more than one *Ulva* species (both *U. mutabilis* and *U. intestinalis*) can respond to the same range of bacteria that affect algal growth, development and morphology via microbial morphogens; (ii) there is specificity in the bacterial signals regulating algal development, e.g. with some bacteria inducing rhizoid formation; (iii) the functions of bacteria (i.e. promoting cell division versus cell differentiation/cell wall formation) cannot be assigned to a specific genus taxonomic group (Fig. 6). This study supports Grueneberg *et al.* (2016) who showed that the presence of specific (epiphytic) bacteria does not directly matter as long as *U. mutabilis* perceives sufficient diffusible morphogenetic compounds even from bacteria in the vicinity of other *Ulva* species within a shared habitat.

Establishing an additional standardised tripartite community (model system) with more than one species of *Ulva* presents an ideal possibility for elucidating the complexity of algal–bacterial partnership. The combined use of the tripartite communities will help to increase understanding of algal growth and development, to shed light on the underlying mechanisms involved in the cross-kingdom cross-talk of algae and bacteria. As *U. intestinalis* is a widespread alga with biofouling properties, our research presents a new way of understanding and controlling the life cycle of an economically important alga.
